# Hypocretins, sleep, and maternal behavior

**DOI:** 10.3389/fnbeh.2023.1184885

**Published:** 2023-06-30

**Authors:** Mayda Rivas, Annabel Ferreira, Pablo Torterolo, Luciana Benedetto

**Affiliations:** ^1^Departamento de Fisiología, Facultad de Medicina, Universidad de la República, Montevideo, Uruguay; ^2^Facultad de Ciencias, Universidad de la República, Montevideo, Uruguay

**Keywords:** hypocretin (orexin), neuropeptide, hypothalamus, medial preoptic area (mPOA), wakefulness, sleep, maternal behavior, lactation

## Abstract

The postpartum period is a demanding time during which mothers experience numerous physiological adaptations that enable them to care for their offspring while maintaining their wellbeing. Hypocretins, also known as orexins, are neuropeptides synthesized by hypothalamic neurons that play a fundamental role in several functions, including the promotion of wakefulness and motivated behaviors, such as maternal care. In this regard, several findings suggest that the activity of the hypocretinergic system increases in the early postpartum period and begins to decline as weaning approaches. In particular, hypocretins within the medial preoptic area, a crucial region during this period, modulate both maternal behavior and sleep. Although further studies are necessary to obtain a comprehensive understanding of the role of hypocretins in lactating females, current research suggests that this system participates in promoting active components of maternal behavior and regulating wakefulness and sleep adjustments during the postpartum period, potentially leading to increased wakefulness during this stage. These adaptive adjustments enable the mother to cope with the continuously changing demands of the pups.

## Introduction

During the postpartum period, multiple modifications in hypothalamic neuronal systems result in physiological adaptations enabling the mother to care for her offspring while preserving her wellbeing (Russell et al., 2001; Numan and Woodside, 2010). The hypocretinergic system, is one of these networks that undergoes functional modifications during this period, suggesting its potential importance in regulating maternal physiology during lactation (Sun et al., 2003; Wang et al., 2003; España et al., 2004; Donlin et al., 2014; Diniz et al., 2018).

Hypocretins (HCRT), also known as orexins, are a pair of peptides (HCRT-1 and HCRT-2, also called orexin A and B, respectively), that originate from the same pre-pro-HCRT (de Lecea et al., 1998). The HCRT bind to two metabotropic receptors, HCRT-R1 and HCRT-R2 (de Lecea et al., 1998; Sakurai et al., 1998), that have both presynaptic and postsynaptic excitatory effects (van den Pol et al., 1998). The neurons that synthesize HCRT are situated in the posterior-lateral hypothalamus and have widespread projections throughout the brain and spinal cord (Peyron et al., 1998).

Distinct populations of HCRT neurons projecting to several areas account for the different functions of this system (Harris and Aston-Jones, 2006). Thus, although initially associated with food intake, HCRT have emerged as key regulators in a diverse range of physiological processes. Their pivotal roles encompass the promotion of wakefulness, modulation of motivated behaviors, and regulation of neuroendocrine and autonomic functions (Ferguson and Samson, 2003; Nishino and Sakurai, 2005; Li and de Lecea, 2020). Additionally, the HCRT system is critical from a medical point of view, as the dysfunction of HCRT neurons is responsible for narcolepsy, an important sleep disorder (Siegel, 1999; Seifinejad et al., 2023).

Despite its significance in motivated behaviors, the role of the HCRT system during the postpartum period has not been extensively studied. Hence, this minireview aims to explore the role of this system during the postpartum period, with a specific emphasis on how it regulates wakefulness and sleep in coordination with maternal behavior and lactation.

## Hypocretinergic system during the postpartum period

Table 1 provides a summary of the main findings related to the hypocretinergic system during lactation. Wang et al. (2003) showed that rats on postpartum day (PPD) 1 have an increase in pre-pro-HCRT mRNA levels compared to rats during pregnancy and on PPD 14. Recent evidence shows an increase in the number of immunoreactive HCRT neurons during lactation on PPD 12 (Sun et al., 2003; Donlin et al., 2014) and on PPD 15 (Diniz et al., 2018) compared to non-lactating animals. This increase occurs specifically in a subpopulation of HCRT neurons located in the anterior part of the posterior hypothalamus (Donlin et al., 2014; Diniz et al., 2018). Conversely, Garcia et al. (2003) found a decrease in pre-pro-HCRT mRNA on PPD 14 in lactating rats compared to proestrus controls. This may be attributed to the specific increase in HCRT levels during the proestrus phase (Porkka-Heiskanen et al., 2004). However, other studies did not observe changes in the mRNA, pre-pro-HCRT immunoreactive neurons, and hypothalamic HCRT levels as determined by radioimmunoassay between lactating animals and controls (Brogan et al., 2000; Cai et al., 2001; Wang et al., 2003; España et al., 2004). These discrepancies between studies may be attributed to differences in the control animals used as well as the methodology employed.

**TABLE 1:** Principal physiological changes of the HCRT system and functions during lactation.

	Animal	References
**Pre-pro-HCRT mRNA levels**		
Increase on PPD 1 compared to PPD 14 and pregnancy	Rat	Wang et al., 2003
No differences on PPD 10 compared to diestrus	Rat	Brogan et al., 2000
No differences on PPD 11–12 compared to postpartum non-lactating animals	Rat	Sun et al., 2003
Decrease on PPD 14 compared to proestrus	Rat	Garcia et al., 2003
**Number of HCRT immunoreactive neurons**		
No differences between lactating on PPD 8 and virgin	Mice	España et al., 2004
No differences on PPD 1, 14, and pregnancy	Rat	Wang et al., 2003
Increase on PPD 12 compared to virgin females in anterior levels	Prairie vole	Donlin et al., 2014
Increase in the number and mean staining intensity neurons on PPD 11–12 compared to non-lactating postpartum rats	Rat	Sun et al., 2003
Increase in an anterior subpopulation on PPD 15 compared to proestrus and diestrus, and decrease between PPD 15 and PPD 21	Rat	Diniz et al., 2018
**HCRT neuronal activity (Fos)**		
Increase on PPD 8 compared to virgin	Mice	España et al., 2004
Increase in the central dorsomedial subpopulation on PPD 15 compared to proestrus and diestrus, and decrease between PPD 15 and PPD 21	Rat	Diniz et al., 2018
**HCRT levels**		
No differences in hypothalamic HCRT-1 or HCRT-2 levels on PPD14 compared to virgin	Rat	Cai et al., 2001
Negative correlation between HCRT-1 levels in the mPOA and the frequency of contact with pups	Rat	Grieb et al., 2018
**HCRT receptors mRNA expression in the hypothalamus**		
Increase in HCRT-R1 on PPD1 compared to PPD14 and pregnant. No changes in HCRT-R2	Rat	Wang et al., 2003
**HCRT effects on maternal behavior and lactation (microinjections)**		
HCRT-1 i.c.v at intermediate doses increases pup-licking and grooming, while high doses reduce pup retrieval, nursing, and nesting behavior	Mice	D’Anna and Gammie, 2006
HCRT-1 into the mPOA increases licking and retrieval, whereas HCRT-R1 antagonist reduces active maternal behavior and promotes nursing	Rat	Rivas et al., 2016
DORA into the mPOA increases nursing and milk ejections	Rat	Rivas et al., 2021
**HCRT effects on postpartum wakefulness and sleep (microinjections)**		
HCRT-1 into the mPOA promotes wakefulness and reduces NREM and REM sleep	Rat	Rivas et al., 2021
DORA into the mPOA increases NREM and REM sleep time	Rat	Rivas et al., 2021
**HCRT effects on prolactin secretion**		
HCRT-1 stimulates prolactin release from lactotroph cells in *in vitro* pituitary explants from lactating animals	Sheep	Molik et al., 2008
**Feeding (mRNA levels)**		
No changes in pre-pro-HCRT after 48 or 72 h of fasting on PPD 14	Rat	Garcia et al., 2003
Increase in HCRT-2 after 48 h of food restriction on PPD 14	Rat	Cai et al., 2001

PPD, postpartum day; mPOA, medial preoptic area; i.c.v, intracerebroventricular.

Related to the HCRT activity during the postpartum period, using *c-Fos* expression as a marker of neuronal activity, two studies found changes during this period. Specifically, España et al. (2004) observed an increase in HCRT neuronal activity on PPD 8 in comparison to cycling virgin mice, whereas in rats, Diniz et al. (2018) found an increase in HCRT activity on PPD15 compared to proestrus and diestrus in a dorsomedial subpopulation. This latter activity was followed by a significant reduction toward the end of lactation (PPD 21). Interestingly, this decrease is modulated by the suckling stimulus of the pups, as interrupting suckling reduces the decline (Diniz et al., 2018).

In summary, these results suggest that the HCRT system is most active during the early postpartum period and that this activity gradually decreases as the postpartum period advances.

Regarding HCRT receptor expression, Wang et al. (2003) reported a significant increase in the mRNA expression of HCRT-R1 in the hypothalamus of female rats on PPD 1 compared to those on PPD 14 and during pregnancy. They also observed an increase in the number of HCRT-R1 immunoreactive cells on PPD 1 in the magnocellular neurons of the supraoptic and paraventricular nuclei, where oxytocinergic neurons are located. However, no significant changes were found in the mRNA expression of HCRT-R2 (Wang et al., 2003). These findings suggest that the expression of HCRT-R1, but not HCRT-R2, is upregulated in the early postpartum period, particularly in putative oxytocinergic neurons.

## Hypocretins regulate maternal behavior

Maternal behavior is crucial for ensuring the survival of offspring by providing warmth, shelter, defense, nourishment, and affect (Stern, 1989). It encompasses a spectrum of behavioral adaptations governed by intricate neuroendocrine mechanisms, enabling the female to effectively meet the demanding of the offspring (Bridges, 2020). Hormonal shifts occurring during late gestation and parturition facilitate the onset of maternal behavior (Bridges, 1984). Once established, maternal behavior is primarily regulated by stimuli originating from the pups (Rosenblatt, 1980).

Given that the HCRT system regulates a range of motivated behaviors (Mahler et al., 2014), it is not surprising that it also affects maternal behavior. In fact, D’Anna and Gammie (2006) demonstrated that the administration of intermediate doses of HCRT-1 into the brain’s ventricles of postpartum mice led to an increase in pup-licking and grooming, while high doses resulted in reduced pup retrieval, nursing, and nesting behavior. Furthermore, they found that the systemic administration of the HCRT-R1 antagonist tended to decrease certain active maternal behaviors while increasing nursing. Hence, optimal levels of HCRT-1 are necessary for the proper display of maternal behavior.

## Hypocretins regulate maternal behavior and lactation through the preoptic area

**FIGURE 1:**
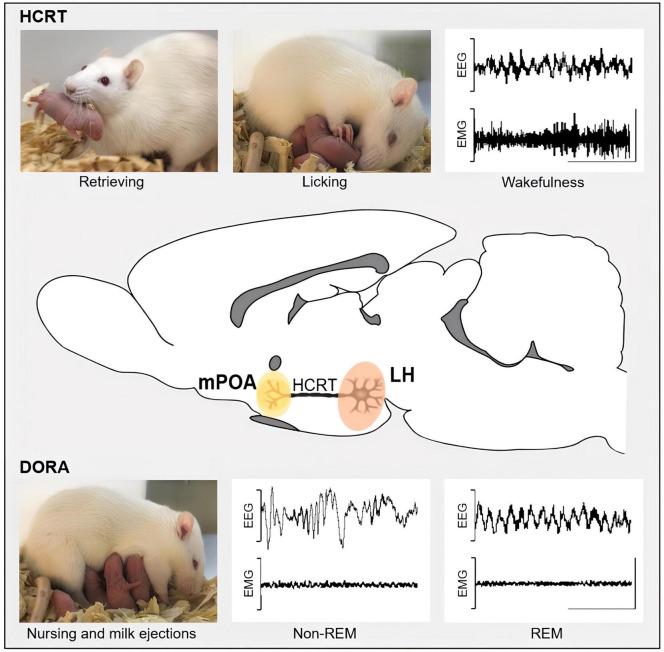
This schematic representation depicts the potential roles of projections from hypocretinergic neurons located in the posterior lateral hypothalamus (LH, highlighted in pink) to the medial preoptic area (mPOA, highlighted in yellow) in the regulation of both sleep and maternal behavior, as demonstrated by intra-mPOA administration of HCRT-1 and the dual hypocretin receptors antagonists (DORA). HCRT-1 intra-mPOA enhances active maternal behaviors, such as licking and retrieval, as well as wakefulness, while DORA promotes nursing along with NREM and REM sleep. EEG, electroencephalogram; EMG, electromyogram. An EEG with high-frequency and low-amplitude waves and an EMG with moderate or high activity characterizes wakefulness. Sleep contains two main stages: REM (rapid eye movement), characterized by high-frequency EEG, with characteristic theta waves in rodents, and muscle atony in EMG; and non-REM sleep, characterized by EEG with low-frequency and high-amplitude waves, and low-activity EMG. Calibration bars: 1 mV and 1 ms.

The medial preoptic area (mPOA) is a crucial brain region where hormones and neuromodulators regulate the maternal care of the pups (Numan, 1974). In our studies, summarized in Figure 1, we found that the microinjections of HCRT-1 into the mPOA of mother rats increased active maternal behaviors such as licking and retrieval, whereas microinjections of an HCRT-R1 antagonist reduced active components of maternal behavior and promoted nursing (Rivas et al., 2016). These results are consistent with those described by D’Anna and Gammie (2006). Together, these findings indicate that the effects of HCRT and the HCRT-R1 antagonist on maternal behavior are mediated, at least in part, by the mPOA. In contrast, HCRT-1 administration in the mPOA did not affect nursing time in our study, whereas D’Anna and Gammie (2006) reported that high doses of HCRT-1 administered intracerebroventricularly, decreased nursing. This discrepancy may be attributed to the different doses and routes of administration used in these studies.

Moreover, the effect of HCRT-1 within the mPOA was more pronounced in the second week of the postpartum period compared to that found in the first week (Rivas et al., 2016). Conversely, the antagonist had a greater effect during the first postpartum week, suggesting that endogenous levels of HCRT-1 are higher in the first compared to the second postpartum week. These findings are consistent with the study of Wang et al. (2003), which reported that hypothalamic levels of pre-pro-HCRT mRNA in lactating rats are elevated on PPD 1 compared to levels observed in the second postpartum week. However, Grieb et al. (2018) did not find variation in HCRT-1 levels within the mPOA analyzed by enzyme immunoassay along the postpartum stage. Nonetheless, the large individual variability found in the study, as well as potential differences between rat strains could account for this discrepancy.

Blocking both HCRT receptors has been found to increase the time that lactating rats spent nursing and in contact with their pups (Rivas et al., 2021). Similarly, Grieb et al. (2018) observed a negative correlation between HCRT levels in the mPOA and the frequency of contact with pups when animals were grouped. Specifically, lower HCRT levels within the mPOA were associated with increased time spent with the pups. This relationship may be due to an increased wakefulness in mothers treated with higher HCRT-1 levels, which may reduce the time they spend in contact with their pups.

Administering HCRT-1 into the mPOA of lactating rats also reduced litter weight gain, despite not affecting nursing time (Rivas et al., 2021). Electrical stimulation of the mPOA has been shown to induce prolactin release (Tindal and Knaggs, 1977), and this area is known to be part of the neural pathway that regulates prolactin release in lactating rats (Tindal and Knaggs, 1977), suggesting that mPOA plays a role in the regulation of prolactin release. Considering that GABAergic neurons in the mPOA inhibit HCRT neurons (Saito et al., 2013), a possible mechanism could be based on the stimulation of the former neurons through intra mPOA administration of HCRT would affect HCRT release, which in turn could reduce prolactin secretion (Fujisawa et al., 2019), resulting in decreased milk production and reduced pup growth. However, as discussed in a following section, prolactin regulation by HCRT is complex and the existing evidence is contradictory.

## Hypocretins regulate wakefulness and sleep during the postpartum period

The behavioral states of sleep and wakefulness with cyclic rhythmicity are well-established in all mammals (Siegel, 2005). Sleep comprises two distinct sleep states known as rapid eye movement (REM) sleep and non-rapid eye movement (NREM) sleep. These behavioral states are accompanied by distinct electrophysiological correlates that enable their identification (Figure 1).

The postpartum period is characterized by significant disruptions in sleep patterns (Stremler et al., 2017). Mothers of diverse species, including humans and rats, exhibit heightened wakefulness to attend to the demands of their offspring, leading to instances of partial sleep deprivation or fragmented sleep (Nishihara et al., 2001; Sivadas et al., 2016; Benedetto et al., 2017; Toth et al., 2020). As HCRT play an essential role in regulating sleep and wakefulness states (Adamantidis et al., 2007; Hung and Yamanaka, 2023), we hypothesized that these peptides contribute to some of the sleep changes that occur during the postpartum period. HCRT neurons have direct projections to critical regions that are involved in generating sleep and wakefulness, such as the thalamus, hypothalamus, and mesopontine reticular (Peyron et al., 1998; Costa et al., 2020). Specifically, projections toward the mPOA may promote wakefulness and active maternal behavior, leading to heightened alertness focused on maternal care of the pups.

While increased levels of HCRT during pregnancy have been linked to poor sleep quality (Qin et al., 2023), the impact of changes in the HCRT system during the postpartum period on maternal sleep remains unclear. Currently, the only study that investigated the effects of HCRT on maternal sleep has focused on its actions through the mPOA (Rivas et al., 2021). In this sense, administering HCRT-1 within the mPOA of lactating rats promotes wakefulness and reduces both NREM and REM sleep (Figure 1), which is consistent with the evidence found in male rats where HCRT-1 had a similar effect (Espana et al., 2001). Furthermore, microinjecting the dual orexin receptor antagonist (DORA) within the mPOA of lactating rats increased both NREM and REM sleep (Figure 1; Rivas et al., 2021).

## Hypocretins regulate wakefulness and maternal behavior through the medial preoptic area

Since lactating rats primarily sleep while nursing (Benedetto et al., 2017), we investigated the concurrent effects of HCRT on wakefulness and sleep, as well as maternal behavior and lactation. The sleep-promoting effects of DORA were accompanied by an increase in nursing time and the number of milk ejections. In contrast, even if the wake-promoting result of HCRT-1 did not lead to changes in nursing time, it still caused a reduction in litter weight gain. As highlighted in Figure 1, these findings suggest a functional integration between sleep, maternal behavior and lactation, regulated by the coordinated actions of HCRT neuronal projections toward the mPOA (Rivas et al., 2021). It could be suggested that HCRT likely acts on specific subpopulations of mPOA neurons to modulate these behaviors. This is supported by extracellular *in vivo* recordings of mPOA neurons in lactating rats, which showed that HCRT-1 increased activity in one group of neurons, while decreasing it in another (Rivas et al., 2022).

## Hypocretins regulate prolactin secretion

HCRT neurons have been implicated in the control of hormone secretion by the anterior pituitary (Taheri, 2006). The effects of HCRT on prolactin synthesis and secretion remain uncertain and subject to debate as limited research has been conducted in the context of lactation. To our knowledge, only one study, conducted by Molik et al. (2008), has investigated the impact of HCRT-1 on prolactin release using *in vitro* pituitary explants from lactating sheep. The results suggest that HCRT-1 directly stimulates prolactin release from lactotroph cells (Molik et al., 2008). On the other hand, in non-lactating females, while there is evidence that HCRT stimulate prolactin release (Kohsaka et al., 2001), it has also been shown that HCRT reduce the expression of this hormone in lactotrope cells (Fujisawa et al., 2019). These findings indicate that the effects of HCRT on prolactin regulation may vary according to reproductive status.

Although there are no functional studies relating HCRT and prolactin, given that prolactin regulates maternal behavior (Bridges, 2020; Georgescu et al., 2021), it could be speculated that HCRT could indirectly influence this behavior through the regulation of prolactin.

Additionally, as prolactin promotes sleep (Roky et al., 1995; Frieboes et al., 1998), HCRT may also modulate sleep indirectly through prolactin regulation. In this sense, the reduction of prolactin expression that results from HCRT administration (Fujisawa et al., 2019), may lead to a decrease in sleep. However, to confirm this hypothesis, further investigation is required.

## Hypocretins may regulate food intake during the postpartum period

To meet the high energy demands of lactation, mothers increase their food intake, which can be partially regulated by HCRT due to its ability to stimulate feeding behavior (Gao and Horvath, 2016). However, Garcia et al. (2003) reported that pre-pro-HCRT mRNA levels in lactating rats on PPD14 did not change after 48 or 72 h of fasting. Conversely, while the expression of pre-pro-HCRT or hypothalamic HCRT-1 levels did not differ between lactating and virgin rats subjected to food restriction or bromocriptine treatment (which inhibits milk production), HCRT-2 levels increased in lactating rats on PPD14 subjected to food restriction (Cai et al., 2001). This finding suggests that HCRT-2 may play a significant role in regulating food intake in lactating rats. Nevertheless, the precise mechanism underlying the hyperphagia during lactation remains unknown.

## Conclusion and future directions

In recent years, there has been a growing interest in studying the HCRT system during the postpartum period. According to the available evidence, the HCRT system increased its activity during the early postpartum period, with levels gradually returning to those of pre-lactation as weaning approaches. These findings suggest a functional role of the HCRT system during this specific period. One function of this system is the promotion of maternal behavior, as optimal brain levels of HCRT-1 are necessary for displaying this behavior correctly. Furthermore, HCRT-1 may play a significant role in regulating wakefulness and sleep adjustment during the postpartum period, potentially leading to increased wakefulness during this stage. These functions are regulated by HCRT, at least in part, through the mPOA. Furthermore, the broad range of functions regulated by the HCRT evidenced in males and cycling females suggests that it may also regulate physiology and behavior during the postpartum period, which has not been fully explored yet. For example, HCRT play an important role in endocrine regulation, indicating that it may participate in hormonal adjustments during lactation.

In addition, to gain a more complete understanding of the role of the HCRT system in lactating females, it would be crucial to conduct studies using advanced techniques such as optogenetics and chemogenetics, to modulate the activity of the HCRT neurons during this period.

Of note, the Food and Drug Administration (FDA) has approved DORA for the treatment of insomnia (Yang, 2014; Scott, 2020), but its effects on lactating mothers and their babies, since this drug has been detected in cord blood and breast milk (Koreki et al., 2023), are still unknown. Therefore, additional research is needed to better understand the impact of these drugs during this critical stage of life, as well as to ensure their safety for use in lactating women.

## Author contributions

MR collected the studies and wrote the first draft of the manuscript. LB, PT, and AF contributed to the writing and provided the critical comments. All authors agreed on the submitted version.
